# Housekeeping genes for quantitative expression studies in the three-spined stickleback *Gasterosteus aculeatus*

**DOI:** 10.1186/1471-2199-9-18

**Published:** 2008-01-29

**Authors:** Sascha Hibbeler, Joern P Scharsack, Sven Becker

**Affiliations:** 1 Department of Evolutionary Ecology, Max Planck Institute for Evolutionary Biology, August-Thienemann-Str. 2, 24306 Plön, Germany; 2 Department of Aquatic Food Webs, NIOO – KNAW Centre for Limnology, Rijksstraatweg 6, 3631 AC Nieuwersluis, The Netherlands

## Abstract

**Background:**

During the last years the quantification of immune response under immunological challenges, e.g. parasitation, has been a major focus of research. In this context, the expression of immune response genes in teleost fish has been surveyed for scientific and commercial purposes. Despite the fact that it was shown in teleostei and other taxa that the gene for beta-actin is not the most stably expressed housekeeping gene (HKG), depending on the tissue and experimental treatment, the gene has been used as a reference gene in such studies. In the three-spined stickleback, *Gasterosteus aculeatus*, other HKG than the one for beta-actin have not been established so far.

**Results:**

To establish a reliable method for the measurement of immune gene expression in *Gasterosteus aculeatus*, sequences from the now available genome database and an EST library of the same species were used to select oligonucleotide primers for HKG, in order to perform quantitative reverse-transcription (RT) PCR. The expression stability of ten candidate reference genes was evaluated in three different tissues, and in five parasite treatment groups, using the three algorithms BestKeeper, geNorm and NormFinder. Our results showed that in most of the tissues and treatments HKG that could not be used so far due to unknown sequences, proved to be more stably expressed than the one for beta-actin.

**Conclusion:**

As they were the most stably expressed genes in all tissues examined, we suggest using the genes for the L13a ribosomal binding protein and ubiquitin as alternative or additional reference genes in expression analysis in *Gasterosteus aculeatus*.

## Background

Recent research has shown that genetic components in gene transcription as well as genetic variability in coding sequences are of evolutionary importance [1,2]. Quantitative reverse transcription polymerase chain reaction (qRT-PCR) is a technique for the quantification of gene expression at the mRNA level that combines the advantages of specificity, sensitivity, speed, throughput and reproducibility. Therefore it is a powerful tool in experimental research [3].

The mRNA level itself is not only influenced by regulation of gene expression. Many other conditions, e.g. nutrition, differences in size and components of the tissue, can influence the mRNA level of the target gene. Therefore it is a straightforward solution to measure the mRNA of the target gene relative to a housekeeping gene (HKG) that is supposed to reflect the status of the fish or tissue and not to be regulated in the selected treatments [4].

In the majority of recent studies either the genes coding for beta-actin (*actb*), 18S rRNA (*18S rRNA*) or glyceraldehyd-3-phosphate dehydrogenase (*gapd*) have been used as reference genes [5]. In the case of the three-spined stickleback, *Gasterosteus aculeatus, actb *was used as the reference gene of choice [2], and until now no other genes have been tested for expression stability and compared with *actb *in this species. Recent studies in teleost fish and other taxa [6-13] suggest that genes cannot be used as a reference gene *per se *without testing their expression stability under the conditions of the desired experiment. Furthermore it has been found that the most stably expressed gene is not necessarily the same in different organs [5]. Therefore the main purpose of this study was to test diverse genes for their suitability as reference genes in different tissues and under different immunological challenges [14,15].

The choice of a suitable reference gene can be a circular problem, because the expression data of this target gene itself also needs to be standardised. One possible solution is to determine the most stable candidate in a group of several genes [16]. Several procedures for this have been suggested, but have rarely been compared. Here we compared 3 different algorithms, implemented in the programs geNorm [15], BestKeeper [16] and NormFinder [17]. We discuss differences in results, taking into account the different approaches to identify the most stably expressed reference gene.

Aim of the present study is to identify reference genes that can be used for expression analysis of immune-relevant genes during an immunological challenge. Therefore our study focused on three immunologically active organs of *G. aculeatus*. First, the head kidney, a major primary and secondary lymphoid organ in teleosts, functioning as a hematopoetic tissue as well as a site for antigen presentation and cell differentiation [18]. Second, the spleen, a major secondary lymphoid organ of teleosts, with essential functions in blood filtering, antigen trapping and processing [18]. Third, the gills were chosen. The gill tissue is in the first line of defence against suspended pathogens in the aquatic environment and therefore is infiltrated with immune cells [19]. For example, Wegner et al. [2] found very high rates of *MHC *(major histocompatibility complex) expression in the gills. We analysed also the gene expression of leukocytes isolated from head kidney and spleen from both a control group and parasite-infected sticklebacks. For this, we used an *in vitro *challenge with parasite antigens and pokeweed mitogen (PWM) as a positive control [20].

Most of the candidate reference genes had not been sequenced before in *G. aculeatus*. Thus, homologous genes in other teleostei were used to find the sequences of the genes in the three-spined stickleback. The homologous genes were aligned with an expressed sequence tag (EST) library and a whole genome shotgun (WGS) library of the three-spined stickleback. Then EST and WGS sequences were aligned to determine exon-intron boundaries for the design of primers that span these regions in the transcripts. In this study we present a method for the reliable measurement of gene expression in *G. aculeatus*.

## Results

### Evaluation of PCR conditions and primers

**Figure 1:**
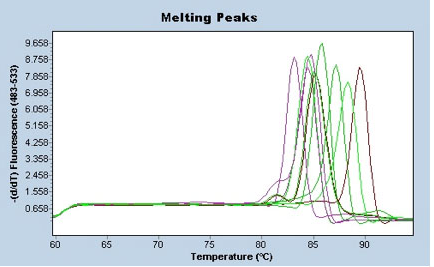
**Dissociation analysis of ten different reference gene PCR products**. The template in all assays was cDNA from the same spleen sample (a whole organ from one fish).

All primer pairs were tested in PCRs with cDNA and gDNA. They were supposed to amplify a single product with a cDNA template, but not to amplify a gDNA template. All primers tested did not amplify gDNA except the primers that target *18S rRNA *(data not shown). The reason for this is that no exon-intron boundaries are present in this target sequence. Consequently, the *18S rRNA *primers amplify both cDNA and gDNA. The application of *18S rRNA *primers to lysates from no-reverse-transcription assays (used as a control) showed that the DNase digestion during mRNA extraction was not always satisfactory (data not shown). Therefore some of the samples contained cDNA and gDNA during PCRs. Furthermore the *ppia *and the *g6pd *gene (see Table 1) were excluded from further analysis, because no product was amplified with primers for these genes during PCR (with cDNA as a template). In order to optimise specificity and efficiency, PCR was tested with various concentrations of our new primer sets, and also a variety of annealing temperatures (60°C – 69°C, data not shown). After optimisation of PCR conditions, amplification products from cDNA were sequenced and checked for their identity in GenBank. This showed that the 10 primer sets that led to amplicon accumulation were stickleback genes. A dissociation analysis was performed after each PCR run with cDNA as a template, in order to show that each primer set amplified the expected single product (Figure 1).

**Table 1:** PCR primers for the amplification of reference gene candidate genes, designed with the software Primer Express 2.0 (Applied Biosystems).

Reference gene		Orientation	5'- 3' Sequence	Bp	Tm (°C)
Beta-Actin	*actb*	For	GCGTGGCTACTCCTTCACC	19	57
		Rev	AGGACTTCATACCGAGGAAGG	21	56
Eucaryotic elongation Factor 1 alpha	*eef1a*	For	CCACCGTTGCCTTTGTCC	18	58
		Rev	TGGGACTGTTCCAATACCTCC	21	57
Hypoxanthine phosphoribosyltransferase 1	*hprt1*	For	GACGCAGATATGGTTCAGATCTC	23	57
		Rev	GTCTTGATTGTGGAGGATATTATCG	25	57
L13A ribosomal binding protein	*rpl13a*	For	CACCTTGGTCAACTTGAACAGTG	23	58
		Rev	TCCCTCCGCCCTACGAC	17	58
RNA-Polymerase II	*rpo2*	For	TTAACAGGTGGGGGGTGC	18	58
		Rev	AGCTCAAGAGCAGAAAGATCCC	22	58
TATA-Box binding protein	*taf2*	For	GGAGTTCATGTTGCAGGTTTTC	22	57
		Rev	CGTTCCTCTTCCTATTGAAGGC	22	58
Ubiquitin	*ubc*	For	AGACGGGCATAGCACTTGC	19	58
		Rev	CAGGACAAGGAAGGCATCC	19	57
Glyceraldehyd-3-phosphate dehydrogenase	*gapd*	For	TGGTCAGCTTACCGTTGAGC	20	58
		Rev	CCCCCTGGCTAAAGTCATCC	20	59
Beta-2-microglobulin	*b2m*	For	AGACTATGCCTGGGAATCAAAC	22	56
		Rev	GAAGATGTGTTGAATAGAAGCTGG	24	56
18S rRNA	*18S rRNA*	For	GACTCCGGTCCTATTTTGTGG	21	57
		Rev	GCTAGTTGGCATCGTTTATGG	21	56
Glucose-6-phosphate dehydrogenase	*g6pd*	For	GGACGGTGTTCCTTTCATCC	20	58
		Rev	TAGGTGAGGTCCAGCTCGG	20	57
Peptidylprolyl isomerase A	*ppia*	For	CCCCTGGCTGGACGG	15	58
		Rev	TAAAAATGACGGGAGGGGG	19	58

### Selection of housekeeping genes

There is no reason to expect a single gene to be the most stably expressed HKG in all tissues [5]. Therefore, data for every organ and every experiment were tested separately, while the data of the different treatments were combined to determine an appropriate reference gene for our study. Even though the three programmes we used presented different orders of the four most stably expressed genes (i.e. *rpl13a*, *actb, ubc *and *gapd*) in cultures of spleen cells (see Table 2), these four selected genes are recognised as stably expressed by all three algorithms used (i.e. Normfinder, geNorm and BestKeeper). The results suggest that all the other genes are less suitable as reference genes. Therefore one of the four selected genes or a combination of them should preferably be chosen as reference genes in cell cultures of spleens. In cultures of head kidney cells the same four genes were selected to be the most stably expressed genes in these experiments (Table 3). There was, however, more disagreement between the programmes concerning the ranking of the reference gene candidates. In whole organs, the differences between the three approaches were more pronounced than in cell cultures. Nevertheless some general observations were made. Two genes, *ubc *and *rpl13a*, were ranked first by at least one of the programmes in each of the three organs. If the experiment is restricted to one of the organs, other candidate genes can be considered as well. In spleens (Table 4) *eef1a *seems to be a reliable candidate gene, while *b2m *might be a good alternative in head kidneys (Table 5). Furthermore, *hprt1 *was ranked second by the programme NormFinder and third by geNorm in gills (Table 6). We encountered failure of single PCR assay series (with primers that target one to three of the ten genes investigated), and we assume that this was related to the accidental omission of primers and/or pipetting errors. Hence, we decided not to include the genes *gapd *and *actb *in the analysis of whole head kidney organs, and *actb *in the analysis of the gills. This was necessary to run the algorithms with sufficient sample numbers, because NormFinder and geNorm require complete data sets, i.e. data from all genes and from the same PCR run. The exclusion of single genes seemed to be justified, because none of them was one of the top candidate genes when taking into consideration the ranking after using the reduced data set: Head kidneys: NormFinder: *actb*, rank 9; *gapd*, 3; geNorm: *actb*, rank 9; *gapd*, 3; BestKeeper: actb rank 8; gapd 6. Results from gills: NormFinder: *actb*, rank 8; geNorm: *actb *rank8; BestKeeper rank 9. (see Additional file 1: Results of reference gene analysis).

**Table 2:** Cultured spleen cells: ranking of the reference gene candidate genes in all samples and within infection treatments, respectively.

	**all (n = 21)**			**Infected/Infected (n = 5)**			**Control/Infected (n = 12)**			**Infected/Control (n = 2)**			**Control/Control (n = 2)**		
**Reference gene**	**Nf**	**gN**	**BK**	**Nf**	**gN**	**BK**	**Nf**	**gN**	**BK**	**Nf**	**gN**	**BK**	**Nf**	**gN**	**BK**

*gapd*	1	1	1	1	1	4	1	1	1	not determined			not determined		
*ubc*	2	4	4	6	6	7	3	6	4						
*rpl13a*	3	3	3	2	1	3	5	5	3						
*actb*	4	1	2	3	5	6	2	3	5						
*rpo2*	5	5	5	9	9	8	4	1	2						
*b2m*	6	6	6	7	7	2	7	7	7						
*eef1a*	7	7	8	4	3	1	8	8	8						
*taf2*	8	8	9	8	8	9	6	4	6						
*hprt1*	9	9	7	5	4	5	9	9	9						

Nf = Normfinder; gN = geNorm; BK = BestKeeper

**Table 3:** Cultured head kidney cells: ranking of the reference gene candidate genes in all samples and within infection treatments, respectively.

	**all (n = 27)**			**Infected/Infected (n = 9)**			**Control/Infected (n = 12)**			**Infected/Control (n = 2)**			**Control/Control (n = 4)**		
**Reference gene**	**Nf**	**gN**	**BK**	**Nf**	**gN**	**BK**	**Nf**	**gN**	**BK**	**Nf**	**gN**	**BK**	**Nf**	**gN**	**BK**

*rpl13a*	1	1	3	5	3	1	3	3	5	not determined			6	1	4
*gapd*	2	6	1	6	4	5	1	1	2				2	7	2
*actb*	3	5	7	4	6	7	8	8	8				4	1	3
*ubc*	4	1	6	8	7	2	4	4	6				1	6	1
*b2m*	5	4	8	1	1	4	2	1	4				5	5	7
*hprt1*	6	7	5	9	9	9	7	7	1				8	3	6
*taf2*	7	8	2	7	8	8	6	6	3				7	8	8
*eef1a*	8	3	9	3	1	3	5	5	9				3	4	5
*rpo2*	9	9	4	2	5	6	9	9	7				9	9	9

Nf = Normfinder; gN = geNorm; BK = BestKeeper

**Table 4:** Whole organs (spleens): ranking of the reference gene candidate genes in all samples and within infection treatments, respectively.

	**all (n = 46)**			**Infected/Infected (n = 13)**			**Control/Infected (n = 10)**			**Infected/Control (n = 10)**			**Control/Control (n = 13)**		
**Reference gene**	**Nf**	**gN**	**BK**	**Nf**	**gN**	**BK**	**Nf**	**gN**	**BK**	**Nf**	**gN**	**BK**	**Nf**	**gN**	**BK**

*ubc*	1	1	2	1	1	3	1	5	3	5	1	3	4	1	3
*rpl13a*	2	1	1	3	3	1	6	6	1	3	1	1	3	1	1
*eef1a*	3	7	5	4	7	5	3	1	4	4	6	4	2	4	5
*rpo2*	4	6	6	2	4	8	4	4	5	1	4	5	8	8	8
*gapd*	5	8	4	7	8	2	5	3	2	8	8	2	1	3	2
*taf2*	6	3	8	8	6	6	2	1	8	6	5	8	5	5	6
*b2m*	7	4	7	6	5	7	7	7	6	2	3	6	7	7	7
*hprt1*	8	5	3	5	1	4	8	8	7	7	7	7	6	6	4
*actb*	9	9	9	9	9	9	9	9	9	9	9	9	9	9	9

Nf = Normfinder; gN = geNorm; BK = BestKeeper

**Table 5:** Whole organs (head kidneys): ranking of the reference gene candidate genes in all samples and within infection treatments, respectively.

	**all (n = 54)**			**Infected/Infected (n = 13)**			**Control/Infected (n = 14)**			**Infected/Control (n = 12)**			**Control/Control (n = 15)**		
**Reference gene**	**Nf**	**gN**	**BK**	**Nf**	**gN**	**BK**	**Nf**	**gN**	**BK**	**Nf**	**gN**	**BK**	**Nf**	**gN**	**BK**

*b2m*	1	3	5	5	5	5	2	3	5	1	4	4	2	4	6
*hprt1*	2	4	6	2	4	4	1	4	4	4	6	6	3	5	5
*ubc*	3	1	1	3	1	1	3	1	3	5	1	1	4	1	1
*eef1a*	4	5	4	4	6	6	5	5	6	3	3	3	1	3	3
*rpo2*	5	6	3	1	1	3	7	7	1	2	5	5	6	6	4
*rpl13a*	6	1	2	6	3	2	4	1	2	6	1	2	5	1	2
*taf2*	7	7	7	7	7	7	6	6	7	7	7	7	7	7	7

Nf = Normfinder; gN = geNorm; BK = BestKeeper

**Table 6:** Whole organs (gills): ranking of the reference gene candidate genes in all samples and within infection treatments, respectively.

	**all (n = 58)**			**Infected/Infected (n = 15)**			**Control/Infected (n = 13)**			**Infected/Control (n = 15)**			**Control/Control (n = 15)**		
**Reference gene**	**Nf**	**gN**	**BK**	**Nf**	**gN**	**BK**	**Nf**	**gN**	**BK**	**Nf**	**gN**	**BK**	**Nf**	**gN**	**BK**

*ubc*	1	1	1	2	1	2	2	1	2	1	1	1	1	1	3
*hprt1*	2	3	6	4	4	6	1	4	5	2	3	6	2	1	5
*rpl13a*	3	1	3	3	1	3	5	1	4	3	1	3	4	3	4
*b2m*	4	4	5	1	3	5	3	3	7	4	4	5	7	4	8
*gapd*	5	6	2	5	5	1	7	6	6	5	6	4	3	6	2
*rpo2*	6	5	8	8	7	7	4	7	3	7	5	8	6	5	6
*taf2*	7	7	7	7	8	8	6	5	1	8	8	7	8	8	7
*eef1a*	8	8	4	6	6	4	8	8	8	6	7	2	5	7	1

Nf = Normfinder; gN = geNorm; BK = BestKeeper

## Discussion

### Primer design and PCR

The PCR primer design for the housekeeping genes (HKG) evaluated in this study faced a general problem. We had to design primers using sequences from North American sticklebacks in order to amplify the cDNA of sticklebacks from our local populations in Germany. Expected sequence differences between the North American and the local stickleback may lead to two problems due to mismatches between primer and target sequence. First, the PCR efficiencies in our assays may differ between target genes. This, however, is monitored with the algorithms used in this study, and thus taken into consideration in all calculations presented. Second, total failure of PCRs may occur. This may explain why it was not possible to amplify all candidate genes of the local stickleback population.

Additional problems may arise from the contamination of the cDNA template with genomic (g)DNA. The minus RT controls of all samples evaluated in this study showed indeed that the DNAse treatment of total RNA did not eliminate the gDNA in every sample. Hence, in some minus RT control qPCR assays we observed a C_P _value, which means that a significant PCR signal arose from gDNA contamination, despite the fact that our primers had a high melting temperature and were designed to span exon-intron boundaries in the transcripts. Due to the comparison of the C_P _values between minus RT control qPCR assays and those assays with cDNA as a template (≤ 5 PCR cycles), we can estimate that the bias introduced by gDNA is a maximum of 3% (data not shown). Therefore primers that are not designed across intron/exon boundaries, and thus amplify gDNA, cannot be used to measure gene expression in our PCR assays. Hence, the *18S rRNA *without introns was not a suitable reference gene in our study *per se*.

The primer concentration in the PCR runs was very high (1,000 nM). This was supposed to outweigh the high annealing/extension temperature of the PCR, because the assay was designed towards specificity rather than efficiency (which after the PCR runs is taken into consideration in the calculations of the three algorithms used). The sensitivity of our PCR assays was demonstrated by the fact that cDNA from 10^5 ^cells was sufficient to measure the expression of 14 genes in parallel PCR runs. The additional purification of mRNA from total RNA was not necessary before reverse-transcription. Hence, the possible loss of mRNA during this additional step did not outweigh the increased efficiency of the following PCRs (data not shown).

It has already been shown that PCR can be inhibited by the enzyme reverse transcriptase, if unpurified cDNA is used as a template [21,22]. Therefore we used a precipitation step to purify the reverse-transcribed cDNA. This also led to improved PCR efficiency (data not shown).

After completion of the thermal programme, all RT-PCRs in this study were subjected to a dissociation analysis to check the identity (by the melting behaviour) of the PCR products. An example for the ten different target gene PCR products from a single cDNA sample is shown in Figure 1. Assays that were not dominated by a single PCR product were excluded from further algorithm analysis. Initially, during optimisation PCR conditions for this study, the PCR products of the new primer pairs were sequenced and checked in GenBank to verify the identity of the target genes. Even though we cannot exclude the amplification of isotypic variants of single genes, our method of gene expression quantification is reliable, because the dissociation analysis would detect high amounts of by-products.

### Housekeeping genes

In this study, we evaluated candidate genes for quantitative real-time PCR assays for immune response gene expression studies in *G. aculeatus*. Unfortunately, there is no universal reference gene that is stably expressed in all tissues and under all biological conditions [3,6-9,12,13]. It is therefore necessary to ensure that no significant regulation occurs during an immunological challenge before a gene can be chosen as standard for relative expression analysis. The potential reference gene should also be stably expressed in tissues with different proportions of lymphocytes and granulocytes as this usually is the case in spleens of natural populations. Therefore we did not separate the different cell types.

Additionally, the chosen gene itself has to be standardised, hence there is a circular problem that needs to be solved. One possible solution is to take more than one gene as the target gene. Pfaffl's solution [16] is to calculate a BestKeeper's index, which is the geometric mean of those genes, that are expressed with a standard deviation (SD) lower than 1. This condition, however, may be too strict, because hardly any of the genes in our cell culture experiments were expressed with a SD lower than 1. In the infection experiments, the SD of the candidate genes was by far too high to include any of them in the calculation of the BestKeeper's index. All other sources of variation, e.g. amount of template, would have to be highly controlled. In the infection experiments, the expression was measured with total RNA from whole organs. This led to a SD too high to calculate a BestKeeper's index according to Pfaffl's conditions. BestKeeper's prerequisites seem to require a very limited experimental setup. Therefore, this algorithm can only be used under limited conditions, whereas a suitable reference gene should be stably expressed under a variety of conditions. Furthermore the quality of each reference candidate gene is determined only by the standard deviation of its expression in different samples. Hence, BestKeeper cannot solve the circular problem outlined above.

The software geNorm [15] suffers from a different problem. In this approach the stability of a candidate gene is determined by pair-wise comparison of variation of expression ratios. Therefore the quality of a reference gene depends on the set of candidate genes that are included in the analysis. The geNorm calculation is only reliable if either most of the other candidate genes are already known to be stably expressed or the candidate genes are not co-regulated. Instead of the most stably expressed gene, geNorm tends to select the gene with the highest degree of similarity to the expression pattern of candidate genes in the whole data set. It has been shown that the elimination of genes from the analysis changed the ranking of the candidate genes [23]. In this study we made similar observations (data not shown).

The NormFinder software [17] combines the advantages of the two other approaches by an estimation of both the intra- and the inter-group expression variation. NormFinder directly and robustly evaluates gene expression stability [23]. It should therefore be preferred to the other two approaches evaluated in this study.

In cell cultures, *rpl13a *and *gapd *seem to be the best reference genes (Table 2 and 3). The other two approaches did not give any reason to exclude *rpl13 *and *gapd *as the best reference gene under these circumstances. *actb *and *ubc *were also good candidates that should be taken into account when more than one reference gene is desired or required.

In the infection experiments, results were less straightforward. In spleens (Table 4) NormFinder suggested *ubc *as the most stable gene. Taking into account the results of all approaches, *rpl13a *is as stable as *ubc*, therefore these two genes should be equally suitable as reference genes.

BestKeeper and geNorm preferred *ubc *and *rpl13a *as the gene of choice for head kidneys (Table 5), but Normfinder suggested that *ubc *should be preferred. In the gills (Table 6) *ubc *and *rpl13a *were the best pair of reference genes. The *hprt1 *may be used as a reference gene in gills and head kidneys as well.

## Conclusion

The study highlights the necessity of pilot studies for selecting the best reference gene(s) in gene expression analysis. Furthermore we can confirm recent studies that question the status of the gene for beta-actin (*actb*) as a general reference gene. Obviously there is no single best gene for all experiments and tissues, even though there seem to be some candidate genes that are stably expressed in a variety of assays and tissues. Our results showed that in most of the tissues and treatments HKG, which could not be used so far due to unknown sequences, proved to be more stably expressed than the gene for beta-actin. As they were the most stably expressed genes in all tissues examined, we suggest using the genes of the L13a ribosomal binding protein and ubiquitin as alternative or additional reference genes in expression analyses in *G. aculeatus*.

## Methods

### Fish for infection experiments

Lab-bred sticklebacks used in infection experiments were full siblings. Experimental fish were about two years old and had been raised as described for family LL5 by Rauch et al. [24]. In May 2006, half of the fish were infected five times with *Diplostomum pseudospathacaeum *(each time 30 parasites per infection and fish), while the control group was not infected. Both the formerly infected group and the control group (35 fish per group) were kept in separate aquaria at 18°C and 16 hours of daylight until the start of the experiment. Before the second round of infection, each fish was transferred to a separate aquarium. The two groups were divided again, and half of each was exposed to 50 individuals of *D. pseudospathacaeum *per fish on 28 July 2006. The other half of the group was not exposed. This resulted in four groups: (1) not exposed in the first and the second round (uninfected control); (2) not exposed in the first round but in the second; (3) exposed in the first, but not in the second round; (4) exposed in both rounds. After 6, 21, 43, and 140 hours three fish of each group were killed by cutting the vertebral column. Groups killed after 260 hours consisted of four fish each. Spleens, head kidneys and gills were dissected from the fish and kept separately in 150 μl of RNA-later (Ambion) for instant preservation of mRNA. The eye lenses were taken from each fish, and living parasites were counted to confirm former infection and to make sure fish were successfully infected during the second round. Recent infections can easily be distinguished from former infections due to the smaller size of the parasites present in the eye lens.

### Cell cultures

Lab-bred fish used in the experiment in May 2006 were full siblings. The parents were wild caught fish from lake Großer Plöner See (Germany). Half of the fish had been infected twice before the dissection. In December 2004, these fish had been infected with 20 individuals of *D. pseudospathacaeum*, 11 *Anguilicola crassus *and 5 *Camallanus lacustris *each. In May 2005, another infection followed with 20 individuals of *D. pseudospathacaeum*, 7 *A. crassus *and 5 *C. lacustris *per fish, while the other half of the fish was a non-infected control group. Cell cultures were prepared from two fish of the control group and two fish that had been infected before. During the dissection the fish were screened for parasites to confirm former infection (data not shown). All steps for cell culture preparation were performed on ice, and only refrigerated media and cooled centrifuges were used. The cell suspensions from head kidneys and spleens were prepared by forcing the tissues through a 40 μm nylon screen (BD-Falcon, USA). Then cells were washed twice (4°C, 10 min 550 × g) with cell medium RPMI 1640 (Sigma-Aldrich) diluted with 10% (v/v) distilled water. Numbers of viable cells (after exclusion of propidium iodide positive cells) were counted by means of flow cytometry: the total cell numbers were determined with a modified form [20] of a standard cell dilution assay (SCDA) [25]. The cell suspensions were adjusted to a density of 4 × 10^6 ^viable cells/ml. Every well (tissue culture test plate 96, Techno Plastic Products) contained 150 μl cell medium [20], 25 μl cell suspension (10^5 ^cells) and 25 μl parasite lysate (3 mg/ml for *D. pseudospathacaeum*, 50 and 5 μg/ml for *C. lacustris*). A positive control [20] was included in the experiment by adding 25 μl pokeweed mitogen (PWM, 16 μg/ml) per well, while the negative control contained 25 μl of cell medium. To obtain parasite lysates, *C. lacustris *nematodes (mainly L4 stages) were collected from intestines of naturally infected sticklebacks, washed in PBS and stored at -20°C. Cercariae of the eye fluke *D. pseudospathaceum *were isolated by placing naturally infected *Lymnaea stagnalis *snails in water-filled (50 ml per snail) glass beakers for 3–5 h under illumination. After sedimentation of suspended matter for 3–6 h in a glass cylinder, water with cercariae was kept at 4°C over night, in order to concentrate the parasite larvae by sedimentation. Subsequently the cercariae were transferred to 50 ml tubes and washed three times with 50 ml PBS by centrifugation (4,500 × g, 4°C, 10 min) before pellets were stored at -20°C. To obtain homogenates, the parasite samples were thawed and adjusted with PBS to 5 ml in a 15 ml tube. The tissue was sonified on ice for 2 × 2 min with a Branson Disruptor Sonofier W-250 (Branson Ultrasonics Corporation, USA; output level 3, duty cycle 50%), solid material removed by centrifugation (4,500 × g, 4°C, 10 min) and the supernatant sterile-filtered, aliquoted and stored at -20°C. Before use, the protein content was determined colorimetrically with a Bradford assay.

The cell cultures were kept at 18°C and 2% CO_2 _(v/v) after set up. After 19 and 40 hours replicates of cell cultures were held on ice for 15 minutes to stop cell activity and detach adherent cells. Then the cell cultures were resuspended and transferred to individual 0.5 ml plastic tubes. An aliquot of 25 μl was used to determine the number of viable cells by means of flow cytometry (see above). The remaining cell suspension was centrifuged at 550 × g for 10 minutes, the supernatant was discarded and the cells were kept in 175 μl of RNA-later (Ambion) for instant preservation of mRNA. All animal experiments described were approved by Ministerium für Landwirtschaft, Umwelt und ländliche Räume (Ministry of Nature, Environment and Rural Development), Bundesland Schleswig-Holstein, Germany.

### RNA extraction and reverse transcription

The RNA was extracted with the NucleoSpin RNA Kit (Macharey-Nagel). Organs were homogenised in tissue lysis buffer with 1% β-mercaptoethanol in a Retsch bead mill by shaking samples for 4 min at 20 Hz. The extraction followed the manual of the kit used, inclusive a DNase digestion step. The extraction from cell cultures was performed with the kit component buffer RA1 (175 μl), 1.75 μl β-mercaptoethanol and 175 μl of ethanol. Total RNA was eluted with two volumes (18 μl) water per sample. All other extraction steps were performed according to the manufacturer's protocol. The synthesis of cDNA was performed with a First-Strand cDNA Synthesis Kit (Amersham Biosciences) according to the manufacturer's protocol. In contrast to the manual of the kit used, the synthesis of cDNA was performed with 2.5 fold of the kit components and 20 μl of the total RNA to increase cDNA yield. The cDNA was precipitated for purification: after the reverse-transcription, 0.75 μl Glycogen (Ambion), 937.5 ng poly dA (Amersham Pharmacia), 2.5 μl sodium-acetate (2 mM, pH 4) and 150 μl cold (-20°C) ethanol (100%) (all molecular biology grade) were added to a total volume of 192 μl according to the protocol by Becker et al [21]. The samples were kept at -20°C over night. On the following day samples were centrifuged at 13,000 × g for 1 hour, followed by removal of the supernatant and an addition of 375 μl cold (-20°C) ethanol (75%). Then the samples were incubated for 10 min at -20°C and centrifuged at 13,000 × g for 15 min. After the removal of the supernatant the samples were incubated at 45°C for at least 15 min to dry the pellet, before it was redissolved in 100 μl nuclease-free water (Sigma). Samples were finally incubated at 45°C for 15 min to enhance pellet redissolution. The cDNA was stored at -20°C until use in quantitative real-time PCR. The remainder of the mRNA was stored at -20°C until it was used as a no-reverse-transcription (-RT) control to check (by PCR) for residuals of gDNA after DNase digestion.

### Primer design and specificity of PCR

All sequences of potential reference genes were retrieved from GenBank. Homologous genes in other teleostei (e.g. *Danio rerio *or *Salmo salar*) were used to find the corresponding sequences in *G. aculeatus*. The homologous sequences were checked (BLAST function in GenBank) against the stickleback sequences in an EST library to find the mRNA sequences. These EST sequences were then checked to find the corresponding sequences in a whole genome shotgun (WGS) library of the three-spined stickleback [26]. Finally, the EST sequences and the sequences of the WGS library of the stickleback were aligned with the program BioEdit to determine exon-intron boundaries. Primers were designed by using the primer analysis software Primer Express 2.0 (Applied Biosystems). One of the primers (synthesised by MWG Biotech, Ebersberg, Germany) of every pair was designed to cover an exon-intron boundary. Since there are no introns in the cDNA, both primers can bind to the cDNA. The introns in the gDNA prevent the primer from binding to the gDNA and therefore from amplifying a PCR product. The desired amplicon length (between 210 and 240 base pairs) was chosen to be similar between all target genes to avoid significant differences in PCR efficiencies due to the amplicon length. The primers were designed with the software Primer Express 2.0 (Applied Biosystems), their specifications are summarised in Table 1.

Before the primers were used for evaluating the expression stability of candidate genes, the identity of the PCR products was checked by sequencing. For this, PCR fragments were generated in assays without Sybr Green, according to the following protocol. PCR assays contained a final volume of 20 μl, including PCR Buffer II (Applied Biosystems), 2 mM MgCl_2_, 500 μM of each dNTP, 0.5 units Ampli Taq Gold (Applied Biosystems), 1 μl (1,000 nM final concentration) of each primer and 2 μl cDNA template. PCR was carried out at an initial incubation for 10 min at 95°C followed by 45 cycles of 20 sec denaturation at 94°C and 1 min annealing/extension at 68°C. 10 μl of the PCR product was mixed with 10 μl SYBR-Green Master Mix (Roche) and dissociation analysis (60°C – 95°C) was performed to check the identity of the amplicon. All amplifications were performed on a LightCycler 480 Instrument (Roche) with a 384-well block. PCR fragments were sequenced to check the identity of the fragments generated with the new primers. For this, 10 μl of the PCR assay described above was purified with the QIAquick PCR Purification Kit (Qiagen), before it was sequenced with the ABI PRISM BigDye Terminator v3.1 Cycle Sequencing Kit (Applied Biosystems), according to the manufacturer's protocol. The cycle sequencing PCR contained a final volume of 10 μl, including 2 μl Cycle Sequencing Mix, 1 μl of both forward and reverse primer (final concentration 1,000 nM), 1 μl buffer, 3 μl water and 2 μl template. PCR was carried out with an initial step of 1 min at 96°C followed by 30 cycles of 10 seconds denaturation at 96°C and 4 min annealing/extension at 60°C. The sequencing of the PCR products with an ABI 3100 capillary sequencer followed the protocol described in Reusch et al. [26]. The sequences obtained were analysed with the software Codoncode-Aligner (Codon Code Company 2000–2004) and were finally checked in GenBank for their identity.

### Real-time PCR

Quantitative real-time PCR was performed on a LightCycler 480 Instrument (Roche) with a 384-well block. Before PCR, the cDNA template of whole organs was diluted 10-fold. Every PCR assay contained a final volume of 20 μl, including 10 μl 2× SYBR-Green Master Mix (Roche), 5 μl diluted cDNA template, 3 μl water and 1 μl (1,000 nM final concentration) of each primer. PCR was carried out with an initial 10 min hot start activation of the polymerase at 95°C followed by 45 cycles of 20 sec denaturation at 94°C and 1 min annealing/extension at 68°C. A dissociation analysis (60°C – 95°C) was performed after completion of the thermal PCR programme to check the identity and purity of the amplification products. All PCR product curves were analysed with the software LinReg PCR [27] to determine the efficiency of every single PCR reaction by linear regression of the exponential section of the product curve, instead of the commonly used ten-fold dilution series and construction of log-linear standard curves. Then N_0 _was calculated with LinReg PCR, which is the input amount of cDNA copies in the reaction before the PCR started. Each PCR run included two no template controls per primer pair, one no reverse transcription control per sample to identify residual gDNA in samples after DNAse digestion, and one positive PCR control.

### Algorithms for selection of reference genes in expression studies

In order to find suitable reference genes for our study, 9 candidate genes that could be amplified with new primers were evaluated (Table 1). The first procedure to estimate the expression stability of target genes was implemented in the software BestKeeper [16]. It calculates the standard deviation of the crossing point (C_P _value) between the whole data set, and the gene with the lowest standard deviation (SD) is proposed most suitable. Every gene with SD higher than 1, is rejected as a reference gene. BestKeeper calculates the C_P _values of the BestKeeper's index. The C_P _values are given as the geometric mean of the remaining gene candidates. Instead of using raw C_P _values for the calculation, BestKeeper was run with the logarithmic N_0 _values according to the programme LinReg PCR (see above), which takes into account the individual PCR efficiency. The second programme evaluated was the software geNorm [15]. The underlying assumption of this programme is that ratios between samples of uniformly expressed, non-normalised target genes should remain regular. GeNorm determines the pair-wise variation between all other target genes as the standard deviation of the logarithmically transformed ratios of expression levels. In iterative steps the gene with the most irregular expression is excluded from further analysis. Therefore the last two remaining genes cannot be ranked. The third programme for selection of reference genes was the software NormFinder [17]. It determines the stability of the candidate genes based on an estimate of the inter- and intra-group variation. It calculates the most stably expressed candidate genes and suggests two of them as references.

## Authors' contributions

SH carried out the experiments and performed the data analysis. He also drafted the manuscript. JPS conducted the cell culture experiment and contributed to the immunological part of the manuscript. SB participated as a supervisor in the study design, data analysis, and editing of the manuscript. All authors read and approved the final manuscript.

## Supplementary Material

Additional file 1 **Results of reference gene analysis**. The file contains detailed results of the three programs and includes additional calculations. Click here for file
